# Evaluation of the effect of carotid sinus blockade on hemodynamic stability in carotid surgery: A retrospective study

**DOI:** 10.1097/MD.0000000000041353

**Published:** 2025-01-24

**Authors:** Dilek Çetinkaya, Ramazan Faruk Bozdoğan, Aykut Şahin, Sadettin Dernek

**Affiliations:** a Department of Anesthesiology and Reanimation, Eskisehir Osmangazi University Medical Faculty, Eskişehir, Türkiye; b Department of Cardiovascular Surgery, Eskisehir Osmangazi University Medical Faculty, Eskişehir, Türkiye.

**Keywords:** carotid endarterectomy, carotid sinus blockade, hemodynamic instability

## Abstract

This study assesses the effect of carotid sinus blockade applied with a local anesthetic on hemodynamic parameters during carotid endarterectomy (CEA) operations performed under general anesthesia. The medical records of patients who underwent CEA under general anesthesia between January 2020 and December 2022, were retrospectively reviewed. It was recorded whether the patients received carotid sinus block with 2 mL of 2% prilocaine. Intraoperative and 48-hour postoperative hemodynamic data were examined in the patients included in the study. A total of 129 patients were evaluated in the study, with 70 patients who received carotid sinus blockade (Group I) and 59 patients who did not receive blockade (Group II) during CEA. The comparison of heart rate variability immediately before clamping, immediately after clamping, and at 5, 10, and 20 minutes post-clamping indicated a significantly greater reduction in Group II compared to Group I (*P* < .05). In the postoperative period, the total dose of glyceryl trinitrate administered was 40.8 ± 31.9 mg in Group I and 53 ± 17.2 mg in Group II, showing a statistically significant difference (*P* = .001). Additionally, blood pressure measurements during this period were significantly higher in Group II than in Group I (*P* < .05). While the application of a local anesthetic during CEA appears to provide better intraoperative heart rate and postoperative blood pressure control, attributing these results solely to local anesthesia may not be entirely accurate. Hemodynamic instability observed during and after CEA is influenced by various factors.

## 1. Introduction

Stenosis or plaque thrombosis and/or fragmentation resulting from atherosclerotic plaques located in the internal carotid artery or carotid bifurcation lead to reduced cerebral perfusion. One of the treatment options for this condition is carotid endarterectomy (CEA), a surgical procedure in which the plaque is removed.^[1]^ The baroreceptors located at the carotid bifurcation play a role in blood pressure regulation.^[2]^ During CEA, surgical manipulations can cause significant bradycardia, which resolves upon cessation of the surgical stimulus. This complicates the surgeon’s work. Another issue encountered is intraoperative blood pressure fluctuations. Postoperative blood pressure instability leads to increased mortality and morbidity. A primary cause of this instability is considered to be the disruption of baroreceptor function due to the removal of the atheromatous plaque during CEA.^[3]^

There are studies in the literature on the application of carotid sinus blockade to address hemodynamic problems during CEA.^[1,3,4]^ However, these studies report varying results regarding the efficacy of carotid sinus blockade applied with local anesthetics.

This study aimed to compare the intraoperative and early postoperative hemodynamic findings in CEA operations performed with and without carotid sinus blockade in our clinic.

## 2. Methods

Following the approval of the local ethics committee (number: 20/February 21, 2023), patients who underwent CEA under general anesthesia between January 1, 2020, and December 30, 2022, were included in the study. Data were obtained from anesthesia records, intensive care follow-up forms, and patient files. Patients who had previously undergone CEA or required reoperation due to postoperative bleeding were excluded from the evaluation.

Patient demographics, including age and gender, the presence of preoperative neurological symptoms, operation side, operation time, and cross-clamp time were recorded. The patients were divided into 2 groups: those who received carotid sinus block during surgery (Group I) and those who did not receive this block (Group II).

All patients underwent intubation following induction with thiopental, rocuronium, and remifentanil, and anesthesia was maintained with remifentanil infusion and sevoflurane. Continuous monitoring included electrocardiography, pulse oximetry, invasive arterial pressure measurement, and regional cerebral oxygenation monitoring using near-infrared spectroscopy (INVOS 5100C Cerebral/Somatic Oximeter, Medtronic, USA).

After routine dissection and heparinization, the carotid artery was clamped and a longitudinal arteriotomy was performed in the problematic area of the carotid artery. The area surrounding the carotid complex and specifically the carotid bifurcation and carotid sinus was exposed. It was the surgeon’s personal choice to perform carotid sinus blockade. Carotid sinus blockade was performed by applying 2 mL of 2% prilocaine around the carotid sinus after exposing the carotid artery.

For both groups, intraoperative (from the time the patient was placed on the operating table until awakening) and early postoperative (first 48 hours) mean arterial pressure (MAP) and heart rate (HR), intraoperative cerebral oxygen saturation for both the right and left sides, and postoperative glyceryl trinitrate dosage were evaluated and compared.

In our institution, vasoactive agents (glyceryl trinitrate for hypertension and norepinephrine for hypotension) are used during CEA operations under general anesthesia when MAP is > 110 mm Hg or < 60 mm Hg, or when cerebral oxygen saturation increases or decreases by 20% from the baseline value. Atropine is administered when HR if < 50 beats per minute.

### 2.1. Statistical analysis

Descriptive statistics of the data included mean, standard deviation, median, minimum, maximum, frequency, and percentage values. The distribution of variables was measured by the Kolmogorov–Smirnov test. The independent-samples *t* test and Mann–Whitney *U* test were used for the analysis of quantitative independent data. The chi-square test was used for the analysis of qualitative independent data. Analyses were performed using SPSS version 28.0.

## 3. Results

A total of 129 patients, out of 132 of whom complete data were available, were evaluated in the study. Among these, 70 patients received a carotid sinus block (Group I), while 59 patients did not receive this block (Group II). The overall mean age of the patients was 70.5 ± 7.1 years, with 45 (34.9%) being female and 84 (65.1%) being male. Group I consisted of 50 males and 20 females, while Group II included 34 males and 25 females. Preoperative neurological deficits were present in 38 (54.3%) patients in Group I and 41 (69.5%) patients in Group II. Surgery was performed on the right side in 55.7% of the patients in Group I and 47.5% of those in Group II. There were no statistically significant differences between the 2 groups in terms of age, gender distribution, the presence of neurological symptoms, or operation side (*P* > .05). In Group I, 43 patients (61.5%) had hypertension, 17 (24.3%) had coronary artery disease, and 23 (32.8%) had diabetes mellitus. In Group II, 38 patients (64.5%) had hypertension, 18 (30.5%) had coronary artery disease, and 24 (40.7%) had diabetes mellitus. The rate of patients with diabetes mellitus was significantly higher in Group II (*P* = .047) (Table 1).

**Table 1:** Demographic data.

		Group I		Group II		*P* value	
		Mean ± SD/n-%	Median	Mean ± SD/n (%)	Median		
Age		70.1 ± 8.1	70.0	71.0 ± 5.9	71.0	.517	*
Gender	Female	20	28.6%	25	42.4%	.101	^X²^ †
	Male	50	71.4%	34	57.6%		
Neurological finding	(-)	32	45.7%	18	30.5%	.077	^X²^ †
	(+)	38	54.3%	41	69.5%		
Operation side	Right	39	55.7%	28	47.5%	.350	^X²^ †
	Left	31	44.3%	31	52.5%		
Comorbidity	HT	43	61.5%	38	64.5%	.565	^X²^ †
	CAD	17	24.3%	18	30.5%	.287	
	DM	23	32.8%	24	40.7%	***.047***	
Operation time (minute)		102.2 ± 20.6	100.0	86.7 ± 11.6	84.0	***.000***	*
Clamp time (minute)		28.8 ± 7.2	28.5	36.6 ± 6.6	36.0	***.000***	*

CAD = coronary artery disease, DM = diabetes mellitus, HT = hypertension, SD = standard deviation.

CAD = coronary artery disease, DM = diabetes mellitus, HT = hypertension, SD = standard deviation.

* Independent-samples *t* test.

† X² = Chi-square test.

The mean operation time in Group I was 102.2 ± 20.6 minutes, which was statistically significantly longer than in Group II, where the mean duration was 86.7 ± 11.6 minutes (*P* = .000). However, the cross-clamp time was longer in Group II, with a mean of 36.6 ± 6.6 minutes compared to 28.8 ± 7.2 minutes in Group I (*P* = .000).

During the intraoperative period, 2 patients in Group I and 3 patients in Group II required atropine for bradycardia, while noradrenaline was administered for hypotension to 5 patients in Group I and 4 patients in Group II.

**Figure 1.**
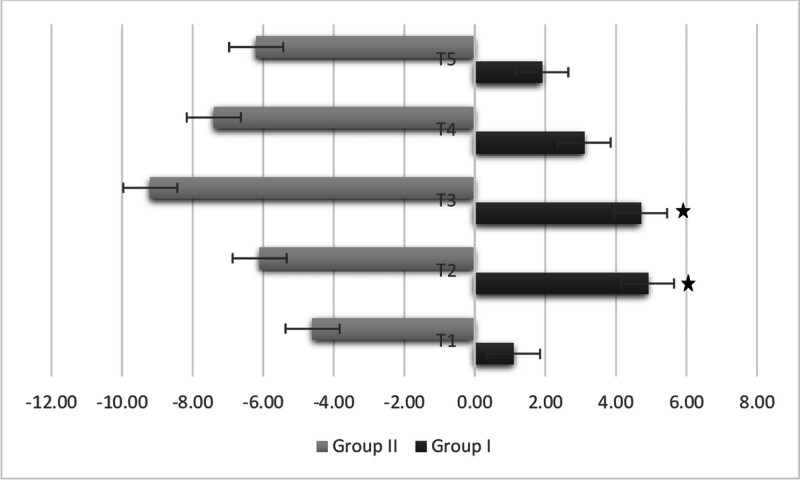
Comparison of heart rate variability between the groups. * Mann–Whitney *U* test (*P* < .05). T1 = post-prilocaine for Group I and pre-clamping for Group II, T2 = at clamping, T3 = 5th minute post-clamping, T4 = 10th minute post-clamping, T5 = 20th minute post-clamping.

**Figure 2.**
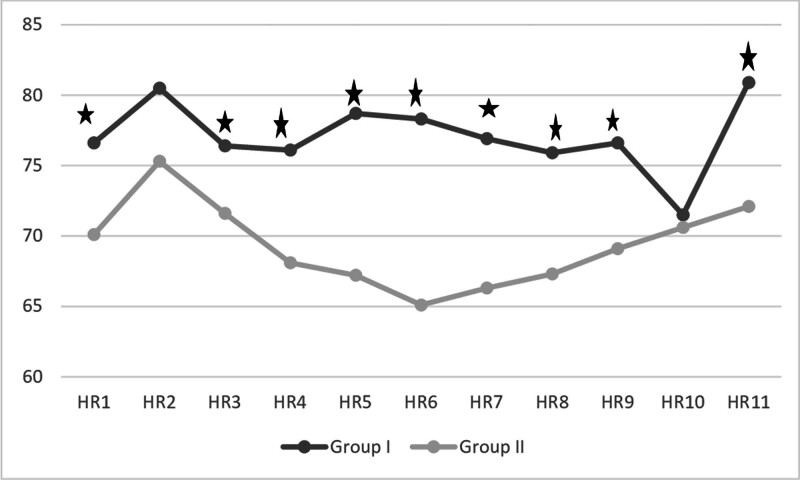
Intraoperative heart rate (HR) values. * Mann–Whitney *U* test (*P* < .05). HR1 = awake, HR2 = post-intubation, HR3 = 15th minute post-intubation, HR4 = post-prilocaine for Group I and pre-clamping for Group II, HR5 = at clamping, HR6 = 5th minute post-clamping, HR7 = 10th minute post-clamping, HR8 = 20th minute post-clamping, HR9 = post-clamp removal, HR10 = immediately before skin closure, HR11 = post-extubation.

When comparing HR variability from the pre-clamp period to immediately after clamping and at 5, 10, and 20 minutes post-clamping, Group II showed a significantly greater negative reduction compared to Group I (*P* < .05) (Fig. 1). Figure 2 presents the intraoperative HR values. In Group II, HR1, HR3, HR4, HR5, HR6, HR7, HR8, HR9, and HR11 values were statistically significantly lower than in Group I. In Group I, there was a significant difference between HR1 (awake) and HR2 (post-intubation), as well as between HR9 (post-clamp removal) and HR10 (immediately before skin closure) (*P* > .05 for both). In Group II, there was a significant difference between HR1 and HR2 (*P* > .05).

Intraoperatively, there were no significant differences between the 2 groups in terms of MAP1 (awake) and MAP5 (at clamping) values (*P* > .05). However, MAP2 (post-intubation) was significantly lower in Group II than in Group I (*P* < .05). In addition, the MAP3 (15th minute post-intubation), MAP4 (post-prilocaine for Group I and pre-clamping for Group II), MAP6 (5th minute post-clamping), MAP7 (10th minute post-clamping), MAP8 (20th minute post-clamping), MAP9 (post-clamp removal), MAP10 (immediately before skin closure), and MAP11 (post-extubation) values were significantly higher in Group II compared to Group I (*P* < .05) (Table 2).

**Table 2:** Mean arterial pressure (MAP) values of the groups.

	Group I (mm Hg)	Group II (mm Hg)	*P* value	
	Mean ± SD	Mean ± SD		
MAP1	111.7 ± 21.4	104.4 ± 10.3	.130	†
MAP2	95.0 ± 24.7	85.0 ± 9.1	.004*	†
MAP3	85.0 ± 15.3	96.8 ± 6.5	.000*	†
MAP4	91.5 ± 14.4	98.8 ± 7.7	.000*	†
MAP5	99.6 ± 12.4	101.6 ± 8.2	.055	†
MAP6	96.8 ± 10.2	100.7 ± 11.1	.022*	†
MAP7	96.5 ± 10.5	101.3 ± 9.9	.008*	†
MAP8	94.8 ± 9.0	100.3 ± 9.2	.001*	†
MAP9	94.1 ± 12.0	98.8 ± 7.3	.001*	†
MAP10	81.3 ± 12.7	96.4 ± 6.7	.000*	†
MAP11	97.2 ± 17.1	109.9 ± 17.6	.000*	†

MAP1 = awake, MAP2 = post-intubation, MAP3 = 15th minute post-intubation, MAP4 = post-prilocaine for Group I and pre-clamping for Group II, MAP5 = at clamping, MAP6 = 5th minute post-clamping, MAP7 = 10th minute post-clamping, MAP8 = 20th minute post-clamping, MAP9 = post-clamp removal, MAP10 = immediately before skin closure, MAP11 = post-extubation.

† Mann–Whitney *U* test.

**Figure 3.**
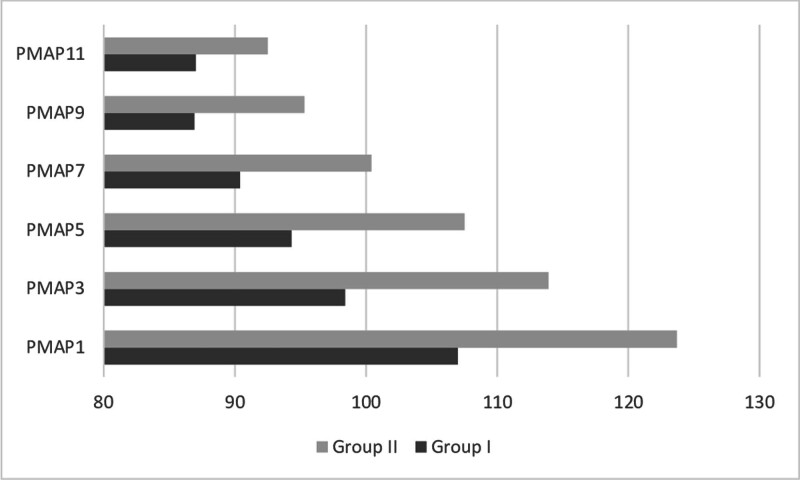
Postoperative mean arterial pressure (PMAP) values of the groups. PMAP1 = initial value in intensive care, PMAP3 = postoperative 2nd hour, PMAP5 = postoperative 6th hour, PMAP7 = postoperative 12th hour, PMAP9 = postoperative 24th hour, PMAP11 = postoperative 48th hour.

In the postoperative period, the total glyceryl trinitrate dose consumed was 40.8 ± 31.9 mg in Group I and 53 ± 17.2 mg in Group II, indicating a statistically significant difference (*P* = .001). Furthermore, the blood pressure measurements in this period were significantly higher in Group II compared to Group I (*P* < .05) (Fig. 3).

## 4. Discussion

This study aimed to compare the hemodynamic data of patients who underwent CEA with and without the administration of a local anesthetic to the carotid sinus. In the group where local anesthetic was not applied, there was greater variability in HR during the intraoperative period following clamp placement, with a tendency toward the slowing of HR. Moreover, higher intraoperative MAP values were observed in this group. In the postoperative period, MAP values and glyceryl trinitrate consumption were higher in the group without local anesthetic application.

The stretching of the carotid sinus region on the internal carotid artery stimulates baroreceptors, which is perceived as hypertension. In response, a decrease occurs in cardiac contractility, HR, and vascular tone.^[3]^ Hemodynamic fluctuations due to baroreceptor damage after CEA are associated with increased cardiovascular mortality and morbidity in the intraoperative period, early postoperative period, and long term. Hypertension and hypotension have been reported in 56% and 50% of patients, respectively.^[5]^

Sigaudo-Roussel et al tested baroreceptor responses in 3 ways to demonstrate baroreceptor damage during CEA. In the first test, a sudden drop in systemic pressure was stimulated following the clamping of the common carotid artery. In the second test, pressure was applied by rubbing the luminal surface of the carotid sinus region 2 different times to stimulate an increase in systemic pressure. The 3rd test was conducted by releasing the clamp and restoring blood flow. The authors observed an increase in blood pressure with minor changes in HR during the first test, indicating a baroreceptor response to occlusion. The rub test performed before plaque removal resulted in a significant decrease in HR and blood pressure, while the post-removal rub test caused a sudden increase in blood pressure. Upon opening the clamp, they found a decline in blood pressure, but HR remained relatively unchanged. In conclusion, they reported that there was partial damage to the baroreceptor mechanism in the carotid artery with this surgery.^[6]^

In addition to the direct damage that occurs during CEA, other factors contributing to baroreceptor dysfunction and hemodynamic instability include preoperative chronic hypertension, diabetes, the effects of antihypertensive medications, and age.^[7]^ In our study, while the number of patients with hypertension and coronary artery disease was similar in both groups, the rate of patients with diabetes mellitus was higher in the group without the block application. This suggests that diabetic neuropathy might contribute to postoperative hypertension in these patients.

Another hypothesis posits that the preoperative stiffness of the carotid artery wall diminishes during endarterectomy, leading to increased sensitivity to pressure-induced tensions and subsequent carotid sinus stimulation, resulting in a drop in blood pressure. On this basis, it has been considered that the application of a local anesthetic can prevent the stimulation of the carotid sinus.^[8]^

A study comparing patients undergoing carotid sinus block with 2 different local anesthetics to those not receiving the block evaluated intraoperative, recovery unit, and intensive care hemodynamic data. The researchers found higher rates of hypertension in the control group in the recovery and intensive care units, while intraoperative hypotension was more frequent in the bupivacaine group, with no significant difference in bradycardia.^[9]^ In another study, researchers applied 1% lidocaine before the removal of the atheroma plaque in CEA and performed intraluminal tension stimulation (rub test) before and after the application. With the stimulation test before injection, they observed a decrease in MAP and HR and an increase in carotid baroreceptor sensitivity. After the lidocaine injection, they found no change in these values and reported that the baroreflex response was eliminated with a local anesthetic injection into the carotid sinus.^[1]^ In the current study, HR values during the cross-clamp period were more stable in the group receiving the carotid sinus block, and MAP values were lower compared to the other group. Similar to the study by Fardo et al postoperative MAP values were found to be higher in the group without local anesthetic application, and glyceryl trinitrate consumption was also higher in this group.^[9]^

A meta-analysis evaluated the results of studies in which carotid sinus blockade was applied with local anesthetics to resolve hemodynamic problems during CEA. The analysis reported that the findings did not support the routine use of local anesthetics. It was emphasized that while baroreceptor dysfunction might develop following CEA, not all patients experienced hemodynamic dysfunction, which could be attributed to the involvement of more than 1 region (e.g., the aortic arch) in the baroreceptor control of blood pressure.^[5]^ Furthermore, it has been observed that significant hypertension occurs after bilateral carotid sinus denervation,^[10]^ and there is greater blood pressure instability following bilateral CEA.^[11]^ Based on these findings, it has been hypothesized that the baroreceptors on the contralateral side may serve as a reserve following unilateral CEA.^[5]^

We included patients who underwent surgery between 2020 and 2022 in our study. The guideline published in 2023 supports the results of our study and routine block is not recommended.^[12]^

Our evaluation revealed that the group that did not receive a local anesthetic experienced minimal change in HR and an increase in MAP after cross-clamp placement, which was expected. However, during surgical manipulations with the clamp, a decrease in HR values was observed, while MAP values remained consistently high, contrary to expectations. Following the removal of the plaque and clamp, the measured MAP readings were lower than anticipated. The group that underwent carotid sinus blockade experienced an elevation in MAP following the application of the local anesthetic and subsequent cross-clamping. Throughout the duration of cross-clamping, there was a more consistent pattern in HR and MAP values. However, both parameters decreased once the cross-clamp was removed.

Our study has several limitations. First, it is a retrospective study, with data obtained from patient records. Second, although the same surgical technique was used, the operations were performed by 2 different teams, with differing cross-clamp times despite similar operation durations. Third, the study included patients with unilateral stenosis, but no standardization was made regarding the presence and degree of stenosis in the contralateral carotid artery. Therefore, the baroreceptor activity on the non-operated side was disregarded.

## 5. Conclusion

The results of this study showed that while intraoperative HR and postoperative blood pressure control were better in the group receiving a local anesthetic, attributing these results solely to local anesthesia is not entirely accurate. Although there is a general impairment in baroreceptor function during and after CEA, various factors contribute to the resulting hemodynamic instability.

## Author contributions

**Conceptualization:** Dilek Çetinkaya.

**Data curation:** Dilek Çetinkaya.

**Formal analysis:** Dilek Çetinkaya, Ramazan Faruk Bozdoğan.

**Funding acquisition:** Dilek Çetinkaya.

**Investigation:** Dilek Çetinkaya, Ramazan Faruk Bozdoğan, Aykut Şahin.

**Methodology:** Dilek Çetinkaya.

**Supervision:** Sadettin Dernek.

**Writing – original draft:** Dilek Çetinkaya, Ramazan Faruk Bozdoğan.

**Writing – review & editing:** Aykut Şahin.
